# 318. Description of Patients Readmitted within 30 Days from COVID-19 Hospitalization

**DOI:** 10.1093/ofid/ofab466.520

**Published:** 2021-12-04

**Authors:** Mahmoud Al-Saadi, Carlos Malvestutto, Mohammad Mahdee Sobhanie, Courtney Hebert, Nora Colburn, Mark Lustberg

**Affiliations:** 1 Ohio State University, Columbus, Ohio; 2 The Ohio State University Wexner Medical Center, Columbus, OH; 3 The Ohio State University College of Medicine, Columbus, OH; 4 OSU Wexner Medical Center, Columbus, Ohio; 5 The Ohio State University, Columbus, Ohio

## Abstract

**Background:**

Severe acute respiratory syndrome coronavirus-2 (SARS-CoV-2) has led to increased hospitalizations and utilization of critical care services. There are few studies describing co-morbidities and demographics associated with patients re-admitted within 30-days of discharge. The purpose of this study is to describe this patient population.

**Methods:**

This was a single-center, retrospective study at The Ohio State University Wexner Medical Center to identify patients who were admitted secondary to SARS-CoV-2 and required readmission within 30 days due to complications that might be associated with COVID-19. Adults admitted between 3/15/2020 and 11/15/2020 were included in this study. Baseline demographics including age, gender and race in addition to select comorbidities were identified.

**Results:**

250 patients were identified who were readmitted for various reasons. Readmitted patients had a median age of 55 years, 44% were male, and 41.2% were Black/African American. 62.4% of the population was obese (BMI ≥30 kg/m^2^) with 21.6% with a BMI ≥ 40 kg/m^2^. The top three co-morbidities seen included Diabetes Mellitus (DM) (32.2%), Hyperlipidemia (48.3%) and Hypertension (51.7%).

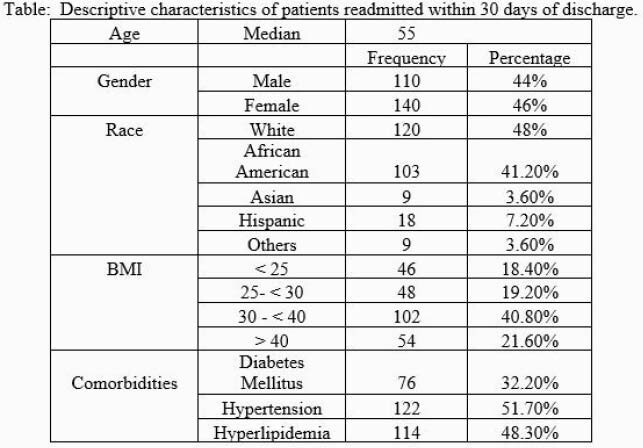

**Conclusion:**

Though this study lacked a comparator group, it is clear that patients readmitted with all cause etiologies were disproportionally Black/African-American and obese, with a high prevalence of DM, hyperlipidemia, and hypertension. We recommend close monitoring of patients in these groups to reduce COVID19 readmissions. This is the first step in identifying which patients may be more likely to develop complications and required readmission, the next step is to compare these patients to those that were not readmitted to develop a risk model for readmission.

**Disclosures:**

**Carlos Malvestutto, M.D.**, **Lilly** (Scientific Research Study Investigator)**Regeneron Inc.** (Scientific Research Study Investigator)**ViiV Healthcare** (Advisor or Review Panel member) **Mohammad Mahdee Sobhanie, M.D.**, **Regeneron** (Scientific Research Study Investigator)**Regeneron** (Scientific Research Study Investigator, Was a sub-investigator for Regeneron 2066 and 2069)

